# Residual Effects of Same Day Lower Extremity Strength Training on Countermovement Jump Performance in Collegiate Women Athletes

**DOI:** 10.5114/jhk/185439

**Published:** 2024-04-25

**Authors:** Bryan L. Riemann, Matthew J. Johnson, George J. Davies, Andrew A. Flatt

**Affiliations:** 1 Biodynamics and Human Performance Center, Georgia Southern University, Armstrong Campus, Savannah, GA, USA.; 2 Department of Health Sciences and Kinesiology, Georgia Southern University, Armstrong Campus, Savannah, GA, USA.

**Keywords:** vertical jump, neuromuscular strategy, athlete monitoring

## Abstract

Balancing of strength programming intensity with sport demands is necessary to avoid excessive workloads that could inhibit performance. To expand previous jump height focused literature, this study evaluated whether countermovement jump (CMJ) movement strategies, including eccentric characteristics, might reveal CMJ execution strategy shifts to achieve similar afternoon CMJ height following a morning resistance training session (RTS). Fifteen collegiate women’s soccer and volleyball athletes (18–24 years, 73.6 ± 8.4 kg, 1.74 ± 0.19 m) participating in an offseason RTS completed five CMJs during two afternoon sessions (48 h apart), one 4–6 h post morning RTS, and one on a rest day. The RTS consisted of 2 sets of 10 repetitions at 70–80% 1RM for the back squat, the front squat, and the forward lunge. Vertical ground reaction forces were recorded from which 13 outcome measures describing elements of the eccentric and concentric CMJ phases were computed. No significant differences in jump height (p = 0.427, d = 0.17) or outcome measures (p = 0.091–0.777, d = −0.07–0.21) between sessions with exception of a significant concentric phase time decrease (p = 0.026, d = 0.23) following the RTS were identified. Given the magnitude of the mean concentric phase time change (0.01 s), the result likely has limited practical meaning. As these results confirm previous CMJ height literature, practitioners have further evidence that a morning RTS does not interfere or enhance afternoon CMJ performance in athletic women.

## Introduction

Studies have examined the effects of various exercises, loads, and volumes on a subsequent neuromuscular performance such as jumping and relevant to many strength and conditioning practitioners is understanding whether there are delayed potentiation effects under the common scenario in which an afternoon competition or practice occurs ~6 h after a morning strength training session. Several studies (Cook et al., 2014; Ekstrand et al., 2013; González-García et al., 2021; Harrison et al., 2021) have sought to examine delayed potentiation effects ~6 h after a resistance priming session. Three of these studies using high load/low volume priming demonstrated statistically significant increases in countermovement jump (CMJ) height (González-García et al., 2021), peak power (Cook et al., 2014), and mean propulsive force (Harrison et al., 2021). Neither the investigation which used a light load/low volume (Saez Saez de Villarreal et al., 2007) nor a high load to failure (Ekstrand et al., 2013) demonstrated significant changes in CMJ performance. One investigation studying changes in CMJ height, anaerobic power, and basketball shooting accuracy 6 h following a moderate intensity exercise session in collegiate female basketball players, more representative of a typical strength training session, reported no significant changes (Woolstenhulme et al., 2004). Thus, aside from overall sport related performance, the effects of a typical morning strength training session on the mechanisms and underlying movement strategies have largely been unstudied.

The CMJ is one of the most common methods of measuring lower extremity strength and power used by coaches, athletes, and researchers (Souza et al., 2020). Jump height (JH) can be readily measured through a variety of inexpensive, simple and practical methods such as jump and reach (Ekstrand et al., 2013), contact mats (Saez Saez de Villarreal et al., 2007; Woolstenhulme et al., 2004), and linear position transducers (Kirby et al., 2011). Unfortunately, the aforementioned methods of assessing JH rely upon a single point on the body to reflect JH rather than a more functional and realistic tracking of the total body center of mass (TBCM). Furthermore, while JH may directly reflect performance ability in sports that involve jumping (Souza et al., 2020), assessing the underlying neuromuscular components contributing to the CMJ via force plate technology provides an enhanced perspective of monitoring training adaptations (Claudino et al., 2017; Gathercole et al., 2015a; Pleša et al., 2022), fatigue (Cormack et al., 2008; Gathercole et al., 2015a), and recovery from musculoskeletal injury (Baumgart et al., 2017).

Despite the CMJ incorporating a stretch shortening cycle preceding the concentric propulsion phase, the majority of CMJ investigations using force plates have focused on the concentric phase with fewer investigations examining characteristics of the eccentric phase (Baumgart et al., 2017; Caserotti et al., 2001; Gathercole et al., 2015a; Harrison et al., 2021). A comprehensive assessment of the underlying CMJ strategies, particularly the eccentric capacity and movement strategies, may better reflect changes in neuromuscular status (Gathercole et al., 2015b; Heishman et al., 2020; Kennedy and Drake, 2017). For example, it was shown that ground reaction force characteristics during the eccentric phase differentiated between patients with anterior cruciate ligament reconstruction (ACLR) with high versus low subjective knee function (Baumgart et al., 2017). Decreased knee eccentric capacity is particularly concerning in ACLR patients as it relates to controlling knee joint translation, flexion, and load absorption during locomotion. Furthermore, changes in eccentric markers of the CMJ, such as eccentric phase duration, were reported to be more affected by acute fatigue than typical concentric measures (Gathercole et al., 2015c).

The aforementioned study (Woolstenhulme et al., 2004) examining the residual effects of a morning full body resistance training session on afternoon CMJ performance is limited as they only considered JH based on a single point estimation. While Woolstenhulme et al. (2004) demonstrated no changes in JH, redundancies within the sensorimotor system could allow an altered movement strategy to achieve similar jump height. Currently, there appears to be an absence of investigations examining whether the eccentric and concentric phase characteristics contributing to CMJ performance may enable an altered CMJ execution strategy to achieve a similar afternoon JH following a morning resistance training session previously reported. Identification of altered movement strategies might provide insight avenues of performance optimization or injury risk. Additionally, the aforementioned study (Woolstenhulme et al., 2004) used a full body resistance training session; whether a morning lower extremity focused training session might induce changes in afternoon CMJ performance remains unknown. Therefore, the primary purpose of this investigation was to examine the residual effects of a same day moderate lower extremity strength training session on concentric and eccentric vertical ground reaction force (VGRF) derived characteristics of CMJ performance in female collegiate athletes. We hypothesized that while jump height would remain unchanged, there would be changes in several of the eccentric and concentric characteristics.

Additionally, investigations have explored the relationships between various CMJ VGRF derived variables and JH. While there is some disparity in results between investigations, generally peak power (Dowling and Vamos, 1993; Markovic et al., 2014; Nuzzo et al., 2008; Sha et al., 2021), peak force (Dowling and Vamos, 1993; Sha et al., 2021), and net vertical impulse (Kirby et al., 2011; Sha et al., 2021) correlate the strongest with JH. The aforementioned investigations monitoring training adaptations, fatigue, and recovery from musculoskeletal injury through force plate derived assessment of CMJ largely used the approach of making comparisons between time-points (e.g., baseline to post-intervention). Particularly for acute training and fatigue interventions, there appears to be a void examining the relationship between changes in CMJ VGRF derived variables and JH. Additionally, except for two studies which conducted analyses across a sample of males and females (Dowling and Vamos, 1993; Peterson et al., 2006), the above referenced investigations considering relationships between CMJ VGRF derived variables and JH used only male participants (Gathercole et al., 2015a; Kirby et al., 2011; Markovic et al., 2014; Sha et al., 2021). Given sex differences in CMJ JH (Kozinc et al., 2021) and CMJ VGRF derived variables, such as concentric impulse, peak power and the center of mass displacement (McMahon et al., 2017), there is a need to study the contribution of various CMJ VGRF derived variables to JH in females. Thus, to determine which VGRF characteristics might be responsible for jump height differences between the two days, a secondary purpose was to examine the association between changes in JH and VGRF characteristics of CMJ performance in female collegiate athletes. We hypothesized that changes in JH would be strongly associated with concentric VGRF characteristics and moderately associated with eccentric VGRF characteristics.

## Methods

### Participants

Participants included fifteen National Collegiate Athletic Association Division I female’s soccer (n = 6) and volleyball (n = 9) athletes (age: 18–24 years, body mass: 73.6 ± 8.4 kg, body height: 1.74 ± 0.19 m ) who were participating in a mid-offseason strength training program. All participants were in good health and were void of significant lower extremity or spinal injuries that prompted a restriction in athletic participation within the past six months. Additionally, all participants were familiar with movements performed in the study as a result of participating in routine strength and conditioning programs. Prior to data collection, participants were given an overview of the study procedures, then signed an informed consent document before completing demographic and health history questionnaires related to musculoskeletal injuries and surgeries. The Institutional Review Board of the Georgia Southern University approved the study (approval code: H06175; approval date: 30 March 2006).

### Measures

During each of the five maximal bilateral countermovement vertical jumps that participants completed at two testing sessions, dual force plates (BP400600NC, Advanced Mechanical Technology, Inc, Watertown, MA) captured ground reaction force data (1000Hz) using the Motion Monitor acquisition software package (Innovative Sports Training, Inc; Chicago IL). The force plates were hardware and software zeroed before each participant. Ground reaction force data were exported as text files and further processed using MatLab (The Mathworks, Inc., Natick, MA) based scripts. First the VGRF data from the two force plates were summed. To avoid the potential for distortion in the velocity and power derived variables (Gathercole et al., 2015a) and “false starts”, visual inspection of the VGRF data from each trial confirmed a quiet stance prior to beginning the CMJ, followed by the manual identification of points just before and after the beginning of the countermovement. Computation of vertical TBCM velocity was conducted beginning at the first point manually identified in the quiet stance (i.e., participant standing stationary) prior to the beginning of the countermovement. The exact beginning of the countermovement was identified by working backwards (Gathercole et al., 2015a) from the second point manually identified to determine the instant when the vertical TBCM velocity <−0.01 m·s^−1^. The transition between the eccentric phase to the concentric phase was defined when the vertical TBCM velocity crossed zero towards positive (Caserotti et al., 2001; Heishman et al., 2019). The eccentric phase was further subdivided into acceleration and deceleration periods based upon when maximal negative vertical TBCM velocity occurred (Caserotti et al., 2001). Ground off was defined when the VGRF < 0.1 N·kg^−1^. Based upon previous reports examining the reliability of various VGRF derived variables (Carroll et al., 2019; Gathercole et al., 2015a; Heishman et al., 2020; Souza et al., 2020), 13 outcome measures describing various elements of the eccentric and concentric phases of the CMJ were computed (Table 1). Forces, power, and impulse were normalized to body weight (Dowling and Vamos, 1993).

**Table 1:** Description of countermovement jump (CMJ) variables.

Variable	Abbreviation	Description
Jump height	JH	Height of the CMJ computed from vertical take-off velocity
Countermovement depth	CMD	Lowest displacement of the total body center of mass
Eccentric phase time	EPT	Length of time for the eccentric CMJ phase
Eccentric acceleration time	EAT	Length of time for the eccentric acceleration period
Eccentric deceleration net impulse	EDNI	Product of average force (body weight removed) during the deceleration period and deceleration period time
Eccentric average deceleration force	EADF	Average force exerted during the deceleration period of the eccentric CMJ phase
Force at zero velocity	F@0V	Force exerted at eccentric to concentric phase transition (i.e., velocity at zero)
Concentric phase time	CPT	Length of time for the concentric CMJ phase
Concentric peak force	CPF	Greatest force exerted during the concentric phase
Concentric average force	CAF	Average force exerted during the concentric phase
Concentric net impulse	CNI	Product of concentric average force (body weight removed) and concentric phase time
Concentric peak power	CPP	Greatest power achieved during the concentric phase
Velocity at peak power	V@PP	Vertical velocity at the instant of peak power during the concentric phase

### Design and Procedures

This study utilized a randomized, cross- over, repeated measures, research design. First, one repetition maximum (1 RM) testing for the barbell back squat, the front squat, and forward lunges was established by a National Strength and Conditioning Association (NSCA) Certified Strength and Conditioning Specialist (CSCS) following the NSCA guidelines (Haff and Triplett, 2016). Forty eight hours later, participants completed one of two afternoon CMJ data collection sessions in random order. One session occurred in the afternoon, 4 to 6 hours after a morning lower extremity resistance training session to replicate the common scenario in which an afternoon practice or game occurs after a morning workout (Woolstenhulme et al., 2004), while the second session occurred on a rest day that did not involve moderate lifting or intensive exercise 36 to 48 h prior (Woolstenhulme et al., 2004). Based upon needing at least 33 hours of recovery time following resistance exercise (Raastad and Hallén, 2000), as well as the common practice of strength and conditioning programs using a Monday-Wednesday-Friday schedule, the two testing sessions were separated by 36 to 48 h. Additionally, to account for diurnal muscle strength fluctuations, the 36- to 48-h interval allowed for testing to occur at the same time of the day. Under the supervision of a NSCA CSCS, the resistance training session consisted of a lower extremity specific resistance training bout including 2 sets of 10 repetitions of the back squat, the front squat and the forward lunge at 70–80% 1RM for a total of 6 sets of 10 repetitions across the three exercises. There was a 2- to 3-min rest interval between sets (Haff and Triplett, 2016).

The testing sessions began with participants first completing a five-minute cycle ergometer warm-up at a Borg Rating of Perceived Exertion (Borg, 1970) between 10 and 12, followed by a standardized lower-extremity dynamic stretching protocol for 5 min. Participants next completed four progressive effort submaximal practice trials, 2 repetitions at 50% perceived effort, 1 at 75% perceived effort and one maximal effort (Carroll et al., 2019; Souza et al., 2020) of an unshod (Benjanuvatra et al., 2013) bilateral vertical CMJ with hands akimbo to minimize arm-swing contribution to vertical jump propulsion (Cormack et al., 2008; Heishman et al., 2019; Impellizzeri et al., 2007). Participants began each CMJ standing stationary with feet shoulder width apart (Impellizzeri et al., 2007). After a 2-s stationary stance period, participants were allowed to self-select countermovement depth (Benjanuvatra et al., 2013; Cormack et al., 2008; Souza et al., 2020). Following the submaximal and maximal trials, participants completed five maximal effort jumps with one minute rest between trials (Benjanuvatra et al., 2013). Verbal encouragement to perform each trial with maximal effort was provided prior to each trial.

### Statistical Analysis

A sample size of 10 was determined to be sufficient based upon a power analysis (α = 0.05, β = 0.2) using the countermovement jump height results comparing control and 80% 1RM training sessions reported by González-García et al. (2021). As the current investigation included additional CMJ derived metrics (i.e., concentric and eccentric vGRF measures), we used a sample size of 15. All outcome measures were averaged across the five trials and the difference scores between the two sessions (a resistance training day vs. a rest day; negative values indicate a measure magnitude decrease for the resistance training day) were examined for normality using QQ plots and Shapiro-Wilk tests. The jump performance variables (Table 1) for each session, were compared with paired *t*-tests. Additionally, standardized effect sizes were computed using Hedges’ g method, adjusted for small samples (Hedges and Olkin, 1985). Following a review of scatterplots, Pearson correlational analysis was conducted between the differences in JH and each of the eccentric and concentric jump performance variables between days. Coefficient magnitude thresholds were interpreted as 0.1, 0.3, 0.5, 0.7, and 0.9 and standardized effect sizes were interpreted as 0.2, 0.6, 1.2, 2.0 and 4.0 for small, moderate, large, very large, and extremely large, respectively (Hopkins et al., 2009). Significance for all inferential statistics was set *a priori* at α < 0.05.

## Results

**Figure 1:**
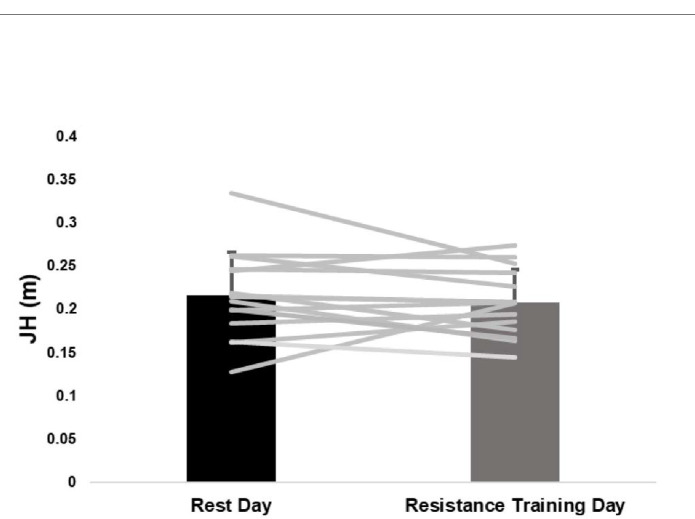
Average and individual jump heights. Error bars are one standard deviation.

**Figure 2:**
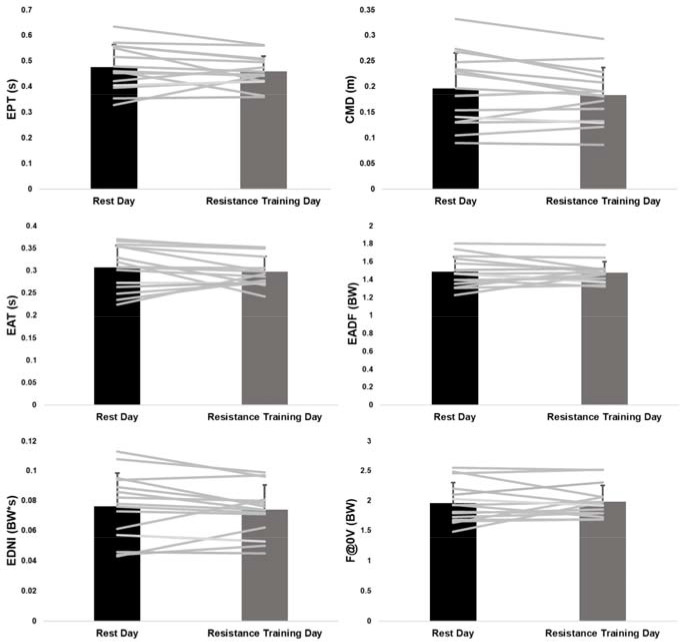
Average and individual values for eccentric variables. Error bars are one standard deviation.

**Figure 3:**
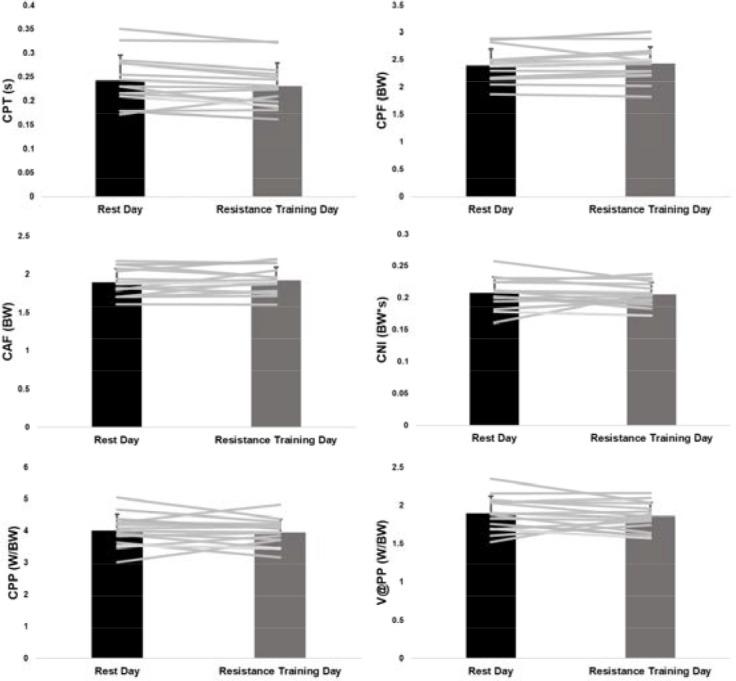
Average and individual concentric variables. Error bars are one standard deviation.

While 66.7% (10/15) of participants demonstrated a decrease in JH on the resistance training day, four exhibited decreases that were less than 0.01 m. As a result, there was no significant difference (*p* = 0.427, *d* = 0.17) in JH between the two days (Table 2). With the exception of CPT (Table 2), none of the outcome measures considered demonstrated significant differences between the rest and the resistance training day. Aside from CPT (*d* = 0.23), 83.3% (10/12) of the effect sizes were < 0.20 and 58.3% (7/12) were < 0.15 suggesting the morning resistance training had minimal effect on afternoon CMJ performance. Individual responses overlaid the group averages between the two sessions are presented for JH (Figure 1), eccentric measures (Figure 2), and concentric measures (Figure 3).

**Table 2:** Results of the paired *t*-tests comparing countermovement jump variables between control and heavy lift days.

Variable	Control Day ($\bar{x}$ ± SD)	Heavy Lift Day ($\bar{x}$ ± SD)	95% CI Difference	*p*	Effect Size
Jump height (m)	0.216 ± 0.050	0.208 ± 0.038	−0.013–0.029	0.427	0.17
Countermovement depth (m)	0.196 ± 0.072	0.183 ± 0.054	−0.223–0 .028	0.091	0.19
Eccentric phase time (s)	0.48 ± 0.08	0.46 ± 0.06	−0.02–0.05	0.283	0.22
Eccentric acceleration time (s)	0.31 ± 0.05	0.30 ± 0.03	−0.01–0.03	0.361	0.21
Eccentric deceleration net impulse (BW·s)	0.076 ± 0.022	0.074 ± 0.016	−0.004–0.008	0.419	0.11
Eccentric average deceleration force (BW)	1.49 ± 0.17	1.48 ± 0.12	−0.06–0.08	0.777	0.06
Force at zero velocity (BW)	1.96 ± 0.34	1.99 ± 0.27	−0.16–0.11	0.700	−0.07
Concentric phase time (s)	0.24 ± 0.05	0.23 ± 0.05	0.002–0.02	0.026	0.23
Concentric peak force (BW)	2.39 ± 0.30	2.43 ± 0.31	−0.10–0.04	0.333	−0.10
Concentric average force (BW)	1.89 ± 0.182	1.92 ± 0.169	−0.09–0.03	0.326	−0.16
Concentric net impulse (BW·s)	0.208 ± 0.024	0.205 ± 0.018	−0.008–0.013	0.591	0.12
Concentric peak power (W·BW^−1^)	4.01 ± 0.50	3.95 ± 0.42	−0.16–0.27	0.569	0.12
Velocity at peak power (m·s^−1^)	1.90 ± 0.22	1.86 ± 0.18	−0.06–0.13	0.437	0.16

SD: standard deviation, CI: confidence interval, BW: body weight

**Figure 4:**
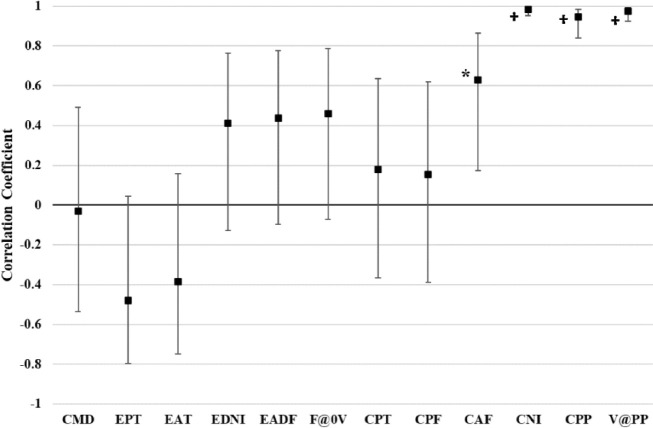
Correlation coefficients bewteen jump height change and each eccentric and concentric variable. Error bars represent 95% confidence intervals for each coefficient. * *p* < 0.05, † *p* < 0.001

There were no statistically significant (*p* > 0.05) associations for the eccentric measures (Figure 4). For the concentric measures, while neither CPT nor CPF differences were associated with JH difference, CAF difference demonstrated a large association (r = 0.63, *p* = 0.012), and CNI (r = 0.98, *p* < 0.001), CPP (r = 0.95, *p* < 0.001), and V@PP (r = 0.98, *p* < 0.001) differences revealed extremely large associations with JH difference.

## Discussion

The primary purpose of this study was to examine the residual effects of a same day lower extremity resistance training session on concentric and eccentric VGRF derived characteristics of CMJ performance in female collegiate athletes. It was hypothesized that the underlying CMJ performance strategies, particularly the eccentric variables, would demonstrate changes in the underlying CMJ strategies to attain JH following a morning resistance training session compared to the strategies exhibited on a rest day. Our results examining afternoon CMJ performance following a morning moderate-high volume lower body resistance training session extend the previous reporting (Woolstenhulme et al., 2004) of no significant changes in afternoon CMJ JH following a morning low volume full body resistance training session. Remarkably, with the exception of a small, but statistically significant decrease in CPT, there were no significant changes in any of the underlying CMJ variables contributing to JH. Additionally, in contrast to our hypothesis, there were no significant changes in the eccentric variables. Thus, by using a comprehensive CMJ performance analysis, the applied meaningfulness of the current results confirms previous work (Woolstenhulme et al., 2004) using a single point estimation of CMJ JH that a morning resistance training session does not promote compelling interference with afternoon CMJ performance in athletic females.

Previous research considering eccentric phase strategies has demonstrated acute changes following induction of neuromuscular fatigue (Gathercole et al., 2015a, 2015c), as well as chronic training responses (Gathercole et al., 2015c). Forces and TBCM velocity at the beginning of the concentric phase are highly influenced by eccentric phase strategies, such as CMD (Sánchez-Sixto et al., 2018), which can subsequently affect JH. While the current statistical results revealed no significant changes in the eccentric CMJ strategies, it is interesting that with the exception of CPT, the effect sizes for EPT, EAT, and CMD were the next largest. Regardless of the small differences in eccentric phase strategies between the days, across all participants, statistically similar JH were attained. If a less optimal eccentric strategy (e.g., longer EAT) was used secondary to residual effects of resistance training, we speculate that subtle compensation could have occurred in other eccentric phase strategies or in the concentric phase to achieve similar JH.

Across all concentric and eccentric measures, the individual response plots demonstrate the greatest between-subjects variability for CMD and EDNI. Previous research has demonstrated that deeper CMD was paired with higher downward TBCM velocities (Sánchez-Sixto et al., 2018). Thus, the variability in CMD likely explains the variability in EDNI needed to bring the downward TBCM velocity to zero. Furthermore, the variability for EADF is lower than EDNI. This suggests that participants likely adjusted the deceleration period duration to accommodate to the varying downward TBCM velocities associated with different CMD. Additional research is needed to better understand the compensatory shifts that occur within the eccentric phase, as well as the concentric phase changes occurring in response to eccentric contribution changes, when CMJs are performed under varying acute (e.g., fatigue) and chronic (e.g., training responses) conditions.

For the concentric variables, the only CMJ variable to demonstrate a significant difference between days was CPT. Of course, it is important to recognize that the mean difference in CPT between sessions was 0.01 s; the applied meaningfulness of such a small change regardless of statistical significance is questionable. As the individual traces in Figure 3 demonstrate, there appears to be a more uniform difference between the two sessions for CPT compared to the other concentric variables which likely explains the statistical significance. Interestingly, while CPT demonstrated a significant decrease, CAF demonstrated a non-significant increase; CNI, the product of CPT and CAF, also demonstrated a non-significant decrease. Notably, the three variables that have been most studied in the CMJ based upon the notion they are closely related to JH, CPP (Dowling and Vamos, 1993; Markovic et al., 2014; Nuzzo et al., 2008; Sha et al., 2021), CPF (Dowling and Vamos, 1993; Kirby et al., 2011; Sha et al., 2021), and CNI (Kirby et al., 2011), were associated with the lowest effect sizes of the concentric variables. These small, non-significant effect results are consistent with the non-significant changes in JH between the two days.

The lack of significant differences between the days is not surprising considering the individual response plots. Examination of the individual response plots illustrates that with the exception of few variables (e.g., CPT, CAF), the majority of participants demonstrated very little change in the other variables between sessions; however, there were a couple of participants who demonstrated larger changes relative to the group majority. It may be plausible that those individuals showing the larger changes may have been more affected, either positively (i.e., potentiation) or negatively (i.e., fatigue), by the resistance training session and thus shifted their CMJ strategies. One explanation for the response variability could be related to differences in strength between those who showed positive versus negative changes. Supporting this notion was a recent report which demonstrated that stronger males experienced CMJ enhancement 24 h after a resistance priming session compared to no CMJ changes for weaker males (Nishioka and Okada, 2022). In addition to showing an increase in JH, improvements in specific eccentric (the rate of force development, mean velocity, mean power, CMD) and concentric (CAF, mean velocity, mean power) measures were documented (Nishioka and Okada, 2022). While this notion cannot be evaluated from the current data, future research should consider whether performance changes following resistance exercise are related to strength in females, as well as include assessments of perceived recovery and soreness scales. The implications of understanding the factors contributing to the resistance training session response variability is relevant for practitioners using the CMJ and other similar metrics to monitor the status of their athletes. For instance, individuals demonstrating substantial decrements in CMJ variables may benefit from modified resistance training loads at morning sessions until they develop a capacity to maintain neuromuscular performance during afternoon practice sessions. Although, such interventions are precautionary until future study determines whether athletes with negatively altered CMJ profiles are at increased risk of injury. Moreover, consistent with previous research (Flatt et al., 2019), the current data could be viewed as supporting the notion that a battery of recovery and response markers reflecting physiological, neuromuscular, and perceived psychological status may be the best approach to evaluating resistance training recovery and adaptation.

The secondary study purpose was to examine the association between changes in JH and VGRF characteristics of CMJ performance as a method to determine which VGRF characteristics might account for JH differences, either increases or decreases, between the two days. In contrast to previous research examining associations between JH and VGRF characteristics using males or mixed sex samples of varying physical activity levels, the current study uniquely focused on a cohort of female collegiate athletes. The extremely large associations clearly indicated that JH changes were closely tied to changes in CNI, CPP, and V@PP. Specifically, if CNI, CPP, and V@PP increased on the resistance training day, JH also increased (and vice versa). These findings support and extend previous reports of strong associations among JH, CPP and CNI in males (Gathercole et al., 2015a; Kirby et al., 2011; Markovic et al., 2014; Sha et al., 2021) and mixed sex samples (Dowling and Vamos, 1993; Peterson et al., 2006) to a cohort of female athletes. While there was a large direct association between changes in JH and CAF, the relationship with CPF difference was small. Whereas one previous investigation demonstrated a small association between JH and CPF (Nuzzo et al., 2008), other investigations (Dowling and Vamos, 1993; Kirby et al., 2011; Sha et al., 2021) have reported moderate to large associations, although uniquely one of the studies (Kirby et al., 2011) reported the relationship to be negative. The association between the changes in JH and CPT was trivial. Again, CNI is the product of CPT and CAF. Given the lack of association with CPT, coupled with large association with CAF, it appears that CAF has a more influential effect on the association between JH changes and CNI than CPT.

Despite the temporal and force correlation coefficients being moderate in magnitude, none of the correlation coefficients between the changes in JH and the eccentric measures were statistically significant. Previously, Dowling and Vamos (1993) demonstrated significant relationships between several eccentric phase measures and JH, and similarly to the current study, the magnitude of the relationships was less than concentric CPP and CPF. Thus, it appears that events of the eccentric phase have lesser influence on JH than the concentric phase (Baumgart et al., 2017; Dowling and Vamos, 1993; Gathercole et al., 2015a), but coupled with previous research, the role of eccentric phase strategies to influence JH remains worthy of further investigation. Of note, the two associations between JH differences and changes in the two eccentric temporal measures, EPT and EAT, were negative indicating that a shorter eccentric-acceleration or overall eccentric phase time were indicative of an increase in JH between the two days. A potential explanation for the inverse relationship is that if CMD remains similar, decreasing EPT and EAT would equate with a higher eccentric velocity, which has been associated with a higher CAF, mean velocity, mean power, and CPF during the propulsive phase compared to slower countermovement velocity (Pérez-Castilla et al., 2019). In contrast, EDNI, EADF, and F@0V were all positively associated suggesting that increases in these measures were associated with an increased JH.

In addition to CPP, CPF and CNI, the influence of CMD on CMJ performance has been the focus of many studies. Studies have demonstrated that deeper CMD results in lower CPF (Kirby et al., 2011; Mandic et al., 2015; Pérez-Castilla et al., 2019; Salles et al., 2011), CPP (Mandic et al., 2015; Pérez-Castilla et al., 2019), and CNI (Kirby et al., 2011). Interestingly, barring one exception (Mandic et al., 2015), the same investigations demonstrated that deeper CMD resulted in higher JH (Kirby et al., 2011; Pérez-Castilla et al., 2019; Salles et al., 2011; Sánchez-Sixto et al., 2018). In contrast to making comparisons to different discrete CMD conditions, Mandic et al. (2015) revealed the association between JH and CMD to be weak. Specifically, changes in JH were less than 0.05 m when CMD varied up to ± 0.20 m from the optimal CMD determined from regression modeling. Our analysis of considering the associations between session changes in CMD and JH is more similar to Mandic et al. (2015) than the studies considering discrete CMD comparisons. Thus, it is not surprising that we obtained compatible results about no association between changing CMD and JH between the two sessions. Similarly to Mandic et al. (2015), we speculate that because of other strategy changes that can occur within the eccentric phase of the CMJ, as well as the concentric phase, this role between CMD and JH during the CMJ is more complicated and requires an investigational approach beyond simple bivariate analysis. Supporting this notion is that when controlling for body mass and CMD, the correlation between CPP and JH became strong using a partial correlational analysis (Markovic et al., 2014). Clearly, there is a need for more investigations into the inter-relationships and eccentric and concentric phase strategy shifts that can occur to produce similar CMJ JH.

There are several limitations of the study. Based upon recruiting NCAA division one female athletes and the complexity of the research design requiring two CMJ assessment sessions following establishment of 1RM on a preceding day, we were limited in study sample size. Given the small between session effect sizes across all variables, it is likely that additional participants would not change the statistical results given the individual response variability. Additionally, during the CMJ, VGRF represented acceleration of the TBCM largely as a result of bilateral ankle, knee, and hip joint contributions to the CMJ. By only examining variables computed from the total VGRF, we cannot determine whether strategy shifts occurred between the limbs or the ankle, knee, and hip joints within a limb. Future research efforts should consider examining the effects of the same day resistance training on limb symmetry and joint kinetics. Furthermore, the study procedures were conducted on two separate days. Thus, not having baseline data for each session is an additional limitation. In addition to inter-day variability in CMJ performance, because each participant completed the CMJ on two separate occasions, there could have been repeated exposure effects (i.e., learning effect, fatigue, etc). However, the randomised cross-over design reduces the chance for systematic bias between the data collection sessions. Additionally, participants could not be blinded to the resistance training day or the rest day, but given the individual variability in CMJ performance between the sessions, there does not appear that consistent bias occurred. Finally, the current study used one resistance training protocol in female soccer and volleyball athletes from one institution; whether these results are generalizable to other protocols (e.g., higher intensity, lower volume, ballistic movements, etc.), males, other sports or levels of collegiate play requires further study.

## Conclusions

In conclusion, this study fills the absence of investigation examining whether the eccentric and concentric phase characteristics contributing to CMJ performance may enable an altered CMJ execution strategy to achieve a similar afternoon JH following a morning resistance training session previously reported. The relevance of filling such void is whether altered movement strategies might provide insight avenues of performance optimization or injury risk. Additionally, given the demonstrated sex differences in CMJ JH and VGRF derived variables, this study contributes to filling the void examining the relationship between changes in CMJ VGRF derived variables and JH in an isolated sample of female athletes.
